# Falling water ice affinity purification of ice-binding proteins

**DOI:** 10.1038/s41598-018-29312-x

**Published:** 2018-07-23

**Authors:** Chen Adar, Vera Sirotinskaya, Maya Bar Dolev, Tomer Friehmann, Ido Braslavsky

**Affiliations:** The Hebrew University of Jerusalem, Robert H. Smith Faculty of Agriculture, Food and Environment, Institute of Biochemistry, Food Science and Nutrition, Rehovot, 7610001 Israel

## Abstract

Ice-binding proteins (IBPs) permit their hosts to thrive in the presence of ice. The ability of IBPs to control ice growth makes them potential additives in industries ranging from food storage and cryopreservation to anti-icing systems. For IBPs to be used in commercial applications, however, methods are needed to produce sufficient quantities of high-quality proteins. Here, we describe a new method for IBP purification, termed falling water ice affinity purification (FWIP). The method is based on the affinity of IBPs for ice and does not require molecular tags. A crude IBP solution is allowed to flow over a chilled vertical surface of a commercial ice machine. The temperature of the surface is lowered gradually until ice crystals are produced, to which the IBPs bind but other solutes do not. We found that a maximum of 35 mg of IBP was incorporated in 1 kg of ice. Two rounds of FWIP resulted in >95% purity. An ice machine that produces 60 kg of ice per day can be used to purify one gram of IBP per day. In combination with efficient concentration of the protein solution by tangential flow filtration the FWIP method is suitable for the purification of grams of IBPs for research purposes and applications.

## Introduction

Ice-binding proteins (IBPs) are produced by many organisms that need to survive in cold environments. These include vertebrates^1^, invertebrates^2,3^, plants^4^, bacteria^5^, and fungi^6^. IBPs have several functions in nature, such as freezing point depression^7^, ice recrystallization inhibition^8^, and ice nucleation^9^. IBPs that function as freezing point depressants are usually termed antifreeze proteins (AFPs). All IBPs bind directly to the surfaces of ice crystals. The ice binding capacity of an IBP grants it the ability to control ice growth and shape, leading to a variety of potential applications in fields that require ice growth control^10^. IBPs are useful in the frozen food industry, agriculture, medicine, biotechnology, and in the cryopreservation of cells, tissues and organs^11–13^. Research studies have shown that IBPs act as cryoprotectants in the cryogenic and hypothermic storage of isolated organs, tissues, cells, sperm, oocytes, embryos, and red blood cells^14–20^. IBPs can also be used to design ice templates in material science and in coatings to prevent ice formation^21,22^. Interestingly, these proteins have not yet been used significantly in most of these industries. The gap between the evident potential of IBPs and their actual applications arises from a lack of sufficient protein quantities and the high cost of the proteins. The availability of large amounts of purified IBPs is indispensable for research, especially in cryopreservation, where low protein quantity hinders the development of practical technologies and limits basic research.

IBPs can be extracted from natural sources (i.e. plants, insects, and fish) or produced by common recombinant technologies^23,24^. Purification can be conducted using standard chromatographic methods. The affinity of IBPs to ice can be used as a purification tool for the selective extraction of IBPs from unpurified solutions. The first ice affinity purification was described by Raymond and Fritsen^25^. Ice active substances were semi-purified from cyanobacteria, green algae and moss crude extracts. The extracts were partly frozen and then centrifuged to partition ice and liquid fractions. Later, two more efficient methods for IBP purification based on ice growth were developed. The basic principle of these methods is that if the ice grows slowly enough, supercooling is minimized, and the engulfment of impurities into the crystal is low. Solution mixing is needed to exclude solutes from the ice^26^. One method is the ice affinity purification technique, in which ice grows slowly on a cold finger immersed in a protein solution. Constant stirring of the solution prevents non-IBPs from the growing ice^26^. The second method is ice shell purification, in which ice is grown on the interior surface of a rotating round-bottom flask submerged in a cool bath^27^. In both methods, an IBP solution in a container is mixed while the temperature is lowered by a programmed water bath. The purified protein is extracted by melting the ice.

In this study, we present a new ice affinity purification method using a commercial clear ice-producing machine. Our method, termed falling water ice affinity purification (FWIP), is an effective, high-yield system. The amount of protein obtained by the FWIP method permits experiments to be performed on the scale of a half liter to hundreds of liters of IBP solution. FWIP is applicable to production of gram quantities of IBP, and thus, can advance IBP research and the use of IBP in cryopreservation, medicine, and other fields.

## Results

### FWIP method

**Figure 1:**
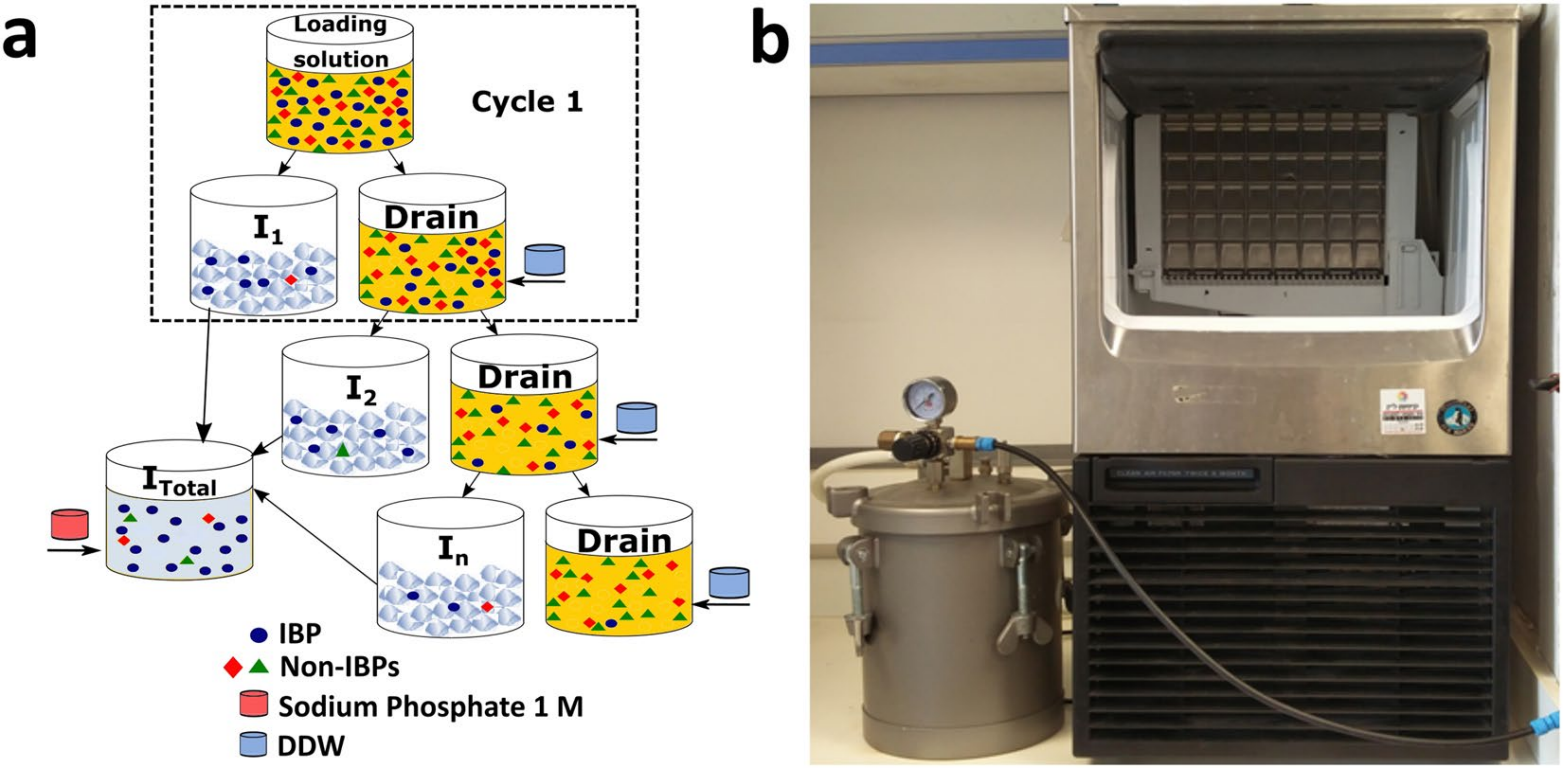
The FWIP process. (**a**) Schematic representation. After one purification cycle, the ice fraction (I_1_) was collected and stored until use. The drain fraction was diluted with double-distilled water (small blue tanks) and was used as the loading solution in the next purification cycle. A series of such cycles is referred here as a round. The ice fractions from the first round (including **n** cycles of FWIP) were mixed (I_Total_) and used as a loading solution in the second FWIP round (not shown). A salt solution (small red tanks) was added to the ice fractions after melting. (**b**) Image of Hoshizaki KM 35A machine and 10 liter Kspark pressure tank that were used for the experiments.

The FWIP method relies on a commercially available ice-making machine (KM 35A, Hoshizaki, Japan) designed to produce clear ice cubes. The machine includes two large metal plates on which ice grows. The metal plates feature columns and rows that produce separate ice cubes. Water supplied to the machine reservoir is elevated by a pump to the top of the metal plates and flows on their surface back to the reservoir. The water is circulated as the temperature of the plates is gradually lowered. Once nucleation occurs below the freezing point, ice begins to grow slowly until the water level decreases or until a time limit is reached. After a batch of ice is produced, the electronic controller initiates a defrost step, the metal plate is heated, and ice cubes drop into the collecting bin. This process constitutes one “cycle” (Fig. 1). At this point, all residual water from the reservoir is drained and another cycle begins.

The gradual temperature drop and the constant water circulation exclude solutes and air bubbles from the growing ice front, yielding clear ice. Any ice-binding substances present are incorporated into the ice. This process is similar to other ice affinity purification methods; however, the metal plate on which the ice grows has a surface area of 900 cm^2^ in the smallest KM machine (model 35A). This surface area is 10 times larger than the surface of a typical cold finger (90 cm^2^ for a finger 10 cm in length). It is also more than twice the surface area of the 1 L round-bottom flasks typically used for ice shell purification^27^. The large surface area provides larger volumes of ice per cycle compared to the cold finger and the ice shell methods. The cold finger and ice shell methods are typically suitable for <1 L volumes, and one cycle requires several hours to complete. After each cycle, the operator must begin the process from the start. By comparison, an ice machine can load 2.5 L within a single 40 min cycle, producing 0.7 L of ice. The machine is designed to work in an automatic mode, repeating freeze cycles one after another. During regular use, a model KM 35 A ice machine consumes 143 L of water and yields 35 kg of clear ice cubes per 24 h without the need for intervention. The ice machine can also be adapted to the purification of smaller volumes. The minimum volume required for one cycle is 0.6 L of loading solution. In this case, a cycle requires only 12 min and produces 0.15 L ice.

We modified the KM machine to allow insertion of a liquid from a pressure tank (Kspark, Taiwan) instead of using water from a regular tap system. Loss of protein via drainage was avoided by collecting the drain liquid after a freezing cycle had completed. This solution was then re-loaded for use in additional purification cycles. The FWIP process is illustrated in Fig. 1. After several purification cycles (a set of such cycles is referred to here as a “round”), all ice was melted and used as the loading solution for a second round of purification. This process yielded ice with a protein purity exceeding 95%. The number of cycles required depended on the volume of the loading solution and the amount of protein in it, and the number of rounds depended on the purification level required.

### FWIP effectiveness in the exclusion of non-IBPs solutes

**Figure 2:**
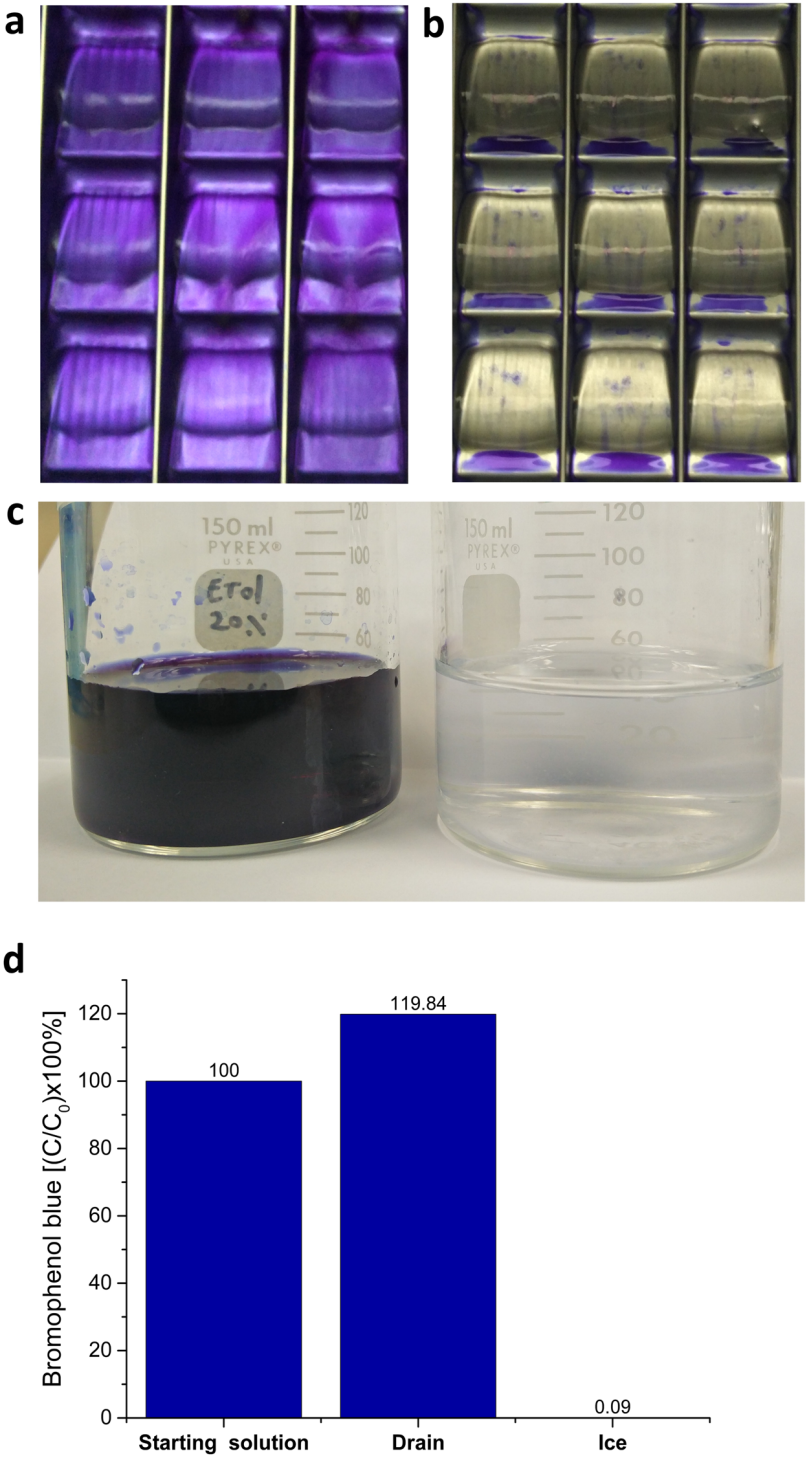
Ice produced by the ice machine excludes non-IBPs solutes. (**a**) A photograph of ice cubes produced in the ice machine using a dye solution. The dye solution runs on top of the growing ice cubes. (**b**) The same cubes in ‘(**a**)’ at the end of the cycle. The observed dye on and under the ice cubes was only external and could be removed by using a dry air flow. (**c**) The drain fraction (left) and the melted ice fraction (right). (**d**) Quantitative evaluation of bromophenol blue in the drain and ice fractions. The dye content was normalized to the starting solution concentration.

**Figure 3:**
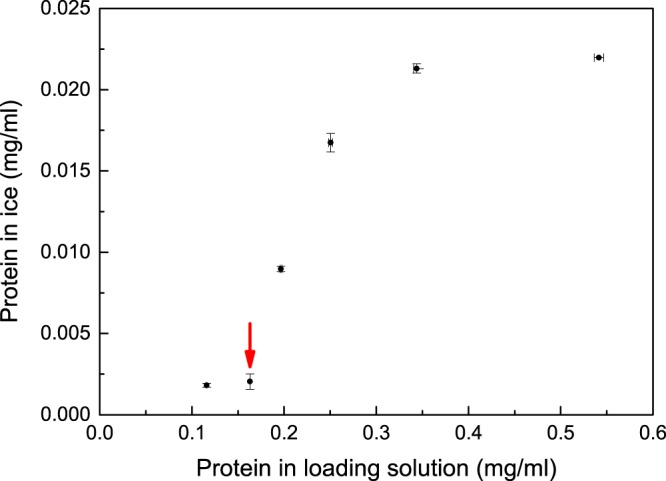
The dependence of impurities in the ice on the total protein (non-IBP) concentration in the loading solution. Each dot represents the average of three independent measurements. The red arrow represents the concentration used for protein purification in subsequent experiments. Non-IBPs were incorporated into the ice at a concentration of 0.002 mg/mL. Standard deviations were calculated from three independent experiments.

The ability of the FWIP method to efficiently exclude solutes from ice was examined. A bromophenol blue solution (0.5 gL^−1^) was run through one cycle of the process, similarly to the examination of the ice shell purification method^27^. Less than 0.1% of the dye, as measured by absorption, was incorporated into the ice fraction, whereas the drain fraction contained concentrated dye (Fig. 2). These results suggested that >99.9% of solutes were efficiently excluded from the ice during a single cycle of FWIP. Purification could be achieved using a relatively dilute starting solution. A loading solution with a high concentration of solutes reduces the purity due to the nonspecific incorporation of molecules into the ice probably due to constitutional supercooling^28^. At the other extreme, a highly dilute loading solution requires more cycles and produces larger volumes that must be handled. The optimal working point that best balances the purity and the dilution factor was determined by purifying lysate solutions of *E. coli* that did not include any IBP plasmids (IBP-free solutions). The total protein concentrations in the loading solution and in the ice fractions were measured. These two values could be described according to a non-linear relationship (Fig. 3). If the total protein concentration in the loading solution exceeded ~0.3 mg/mL, the protein concentration in the ice reached 0.02 mg/mL. At a starting concentration of 0.16 mg/mL or less, the ice obtained was considerably clean, with only 0.002 mg/mL protein. This finding suggested that a starting solution with a total protein concentration of ~0.16 mg/mL yielded ice that was adequately pure without requiring further dilution.

### Amount of IBP incorporated into the ice

**Figure 4:**
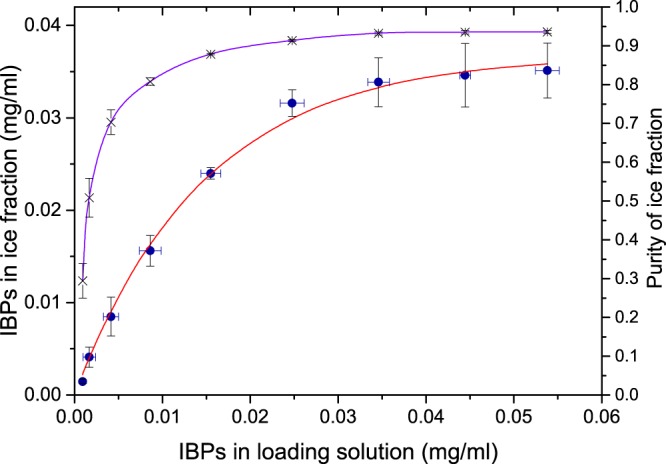
The IBP concentration in ice and the ice purity as a function of the IBP concentration in the loading solution. () The concentrations of the type III AFP in the ice, the data represent the average results obtained from the experiments presented in Fig. S1. (**X**) The purity was determined as the concentration of IBP relative to the concentration of the total protein in the ice fraction as described in equation (2). Standard deviations are indicated by the black bars.

We next quantified the amount of protein that could be purified using the FWIP method as a function of the IBP and impurity content in the loading solution. To this end, we conducted three types of experiments. In the first, we loaded a solution of pure type III AFP without impurities. In the second, we added pure type III AFP to a solution containing 0.16 mg/mL lysate proteins. In the third, we used a crude *E. coli* lysate containing type III AFP diluted to 0.16 mg/mL total lysate proteins. We found that the amount of IBP extracted in each of these experiments depended on the IBP concentration in the loading solution but not on the concentration of the impurities (Supplementary Fig. S1). The concentration of IBPs in the ice increased as a function of the IBP concentration in the loading solution (Fig. 4). This correlation could be expressed by the following equation:

1 $${\rm{I}}{\rm{B}}{\rm{P}}\,{\rm{i}}{\rm{n}}\,{\rm{i}}{\rm{c}}{\rm{e}}=0.037\,{\rm{m}}{\rm{g}}/{\rm{m}}{\rm{L}}\ast (1-{e}^{-\frac{{\rm{I}}{\rm{B}}{\rm{P}}{\rm{i}}{\rm{n}}{\rm{s}}{\rm{o}}{\rm{l}}{\rm{u}}{\rm{t}}{\rm{i}}{\rm{o}}{\rm{n}}}{0.015{\rm{m}}{\rm{g}}/{\rm{m}}{\rm{L}}}})$$

Thus, the amount of IBPs that could be extracted in a cycle reached a maximum value. Further increases in the concentration of proteins in the loading solution did not increase the amount of IBPs present in the ice. The concentration of non-IBPs in the ice fraction remained constant in each experiment because the dilution and volume remained constant in all FWIP cycles. The constant impurity concentration was verified by SDS-PAGE (Supplementary Fig. S2).

The impurity levels in the ice fractions prepared with IBPs were shown to agree with the results obtained in ice fractions prepared without IBPs using data from an experiment in which a known quantity of type III AFP and a known quantity of impurities were present in the loading solution. The quantity of impurities present in the ice was the same as that obtained in the absence of IBPs, 0.002 mg/mL. Using this result, we calculated the protein purity in the ice according to the following equation:

2 $${\rm{Purity}}=({\rm{IBP}}\,{\rm{in}}\,{\rm{ice}})/({\rm{IBP}}\,{\rm{in}}\,{\rm{ice}}+0.002\,{\rm{mg}}/{\rm{mL}})$$

**Figure 5:**
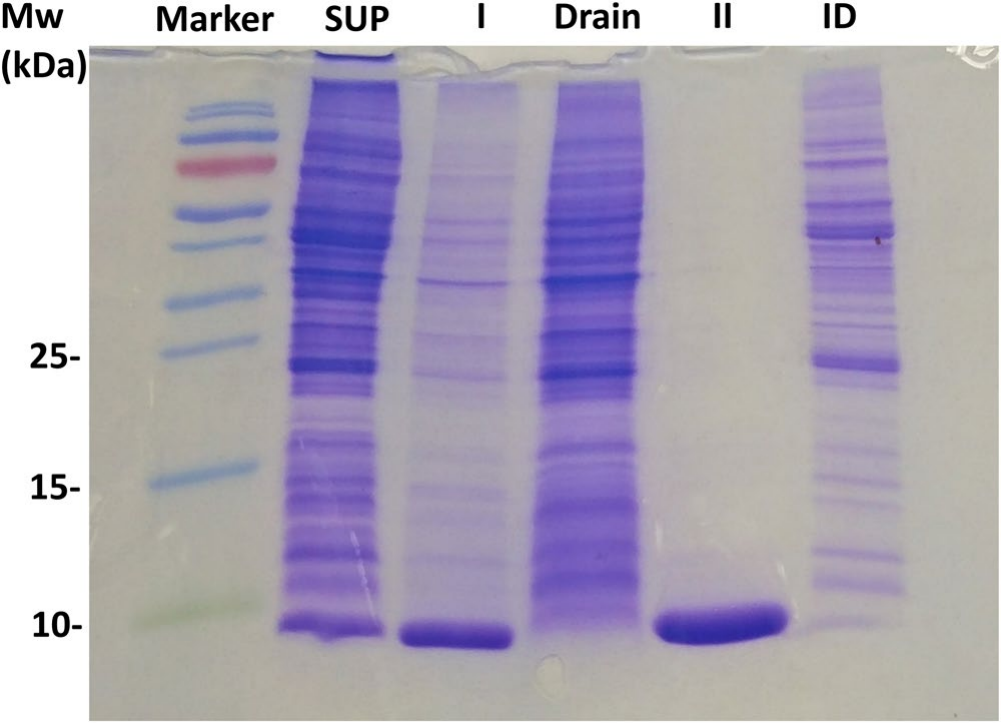
SDS-PAGE analysis of a type III AFP purified by FWIP. From left to right: marker (molecular weight noted on the left); *E. coli* crude lysate supernatant before FWIP (SUP); combined ice fractions obtained from the first purification round (I); liquid fraction obtained from the first purification round (Drain), combined ice fractions obtained from the second purification round (II); liquid fraction obtained from the second purification round (ID). The round II sample concentration was twice the concentration of the other samples, indicating the purity of the protein.

Figure 4 shows that the protein purity was 95% in the first 4–5 cycles of the first round of FWIP. In later cycles, the purity gradually decreased due to a reduction in the IBP concentration in the solution (and, consequently, in the ice) whereas the impurity level remained constant. We were able to extract >99% of the protein present in the crude lysate over 7 cycles of FWIP. Figure 5 presents SDS-PAGE results obtained from the type III AFP following two rounds of FWIP. After the second round of purification, the protein appeared as a pure single band.

### Purification of a variety of IBPs by FWIP

**Figure 6:**
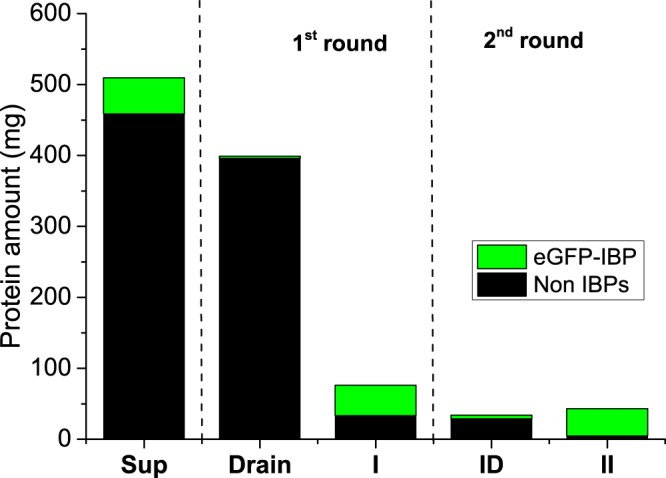
eGFP-*Ri*AFP quantities present in the ice and liquid fractions following FWIP. Non-IBP quantities are shown in black, and eGFP-*Ri*AFP quantities are shown in green. All samples were concentrated to an equal volume. I and II indicate the combined ice fractions obtained from the first and second purification rounds, respectively. ID - drain from the second purification round. Drain - drain from the first purification round.

**Figure 7:**
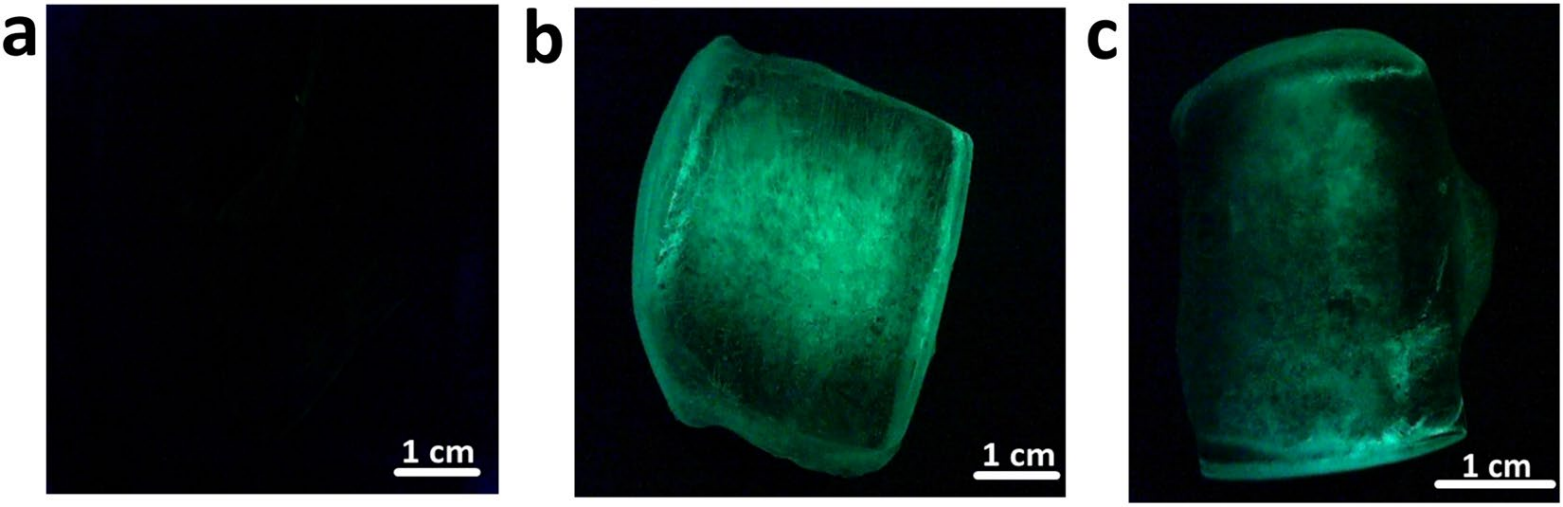
Fluorescent signal obtained from ice cubes prepared during FWIP. (**a**) Control - ice cube prepared from an IBP-free bacterial lysate loading solution. (**b**) Ice cube prepared from a first purification cycle applied to the eGFP-*Ri*AFP. (**c**) Ice cube obtained from the fifth purification cycle applied to eGFP-*Ri*AFP. All cubes were obtained during the first purification round.

We evaluated the ability of the FWIP method to purify three other types of IBPs as pure IBPs or as fusion proteins: AFP from *Rhagium inquisitor* fused to green fluorescent protein (eGFP-*Ri*AFP), recombinant *Tenebrio molitor* AFP (*Tm*AFP) fused to maltose binding protein (MBP-*Tm*AFP), and *Tm*AFP extracted from *Tenebrio molitor* larvae. We conducted 4–6 consecutive cycles as a first round of FWIP and collected the ice for a second round of purification (additional 4–6 cycles). The purities of the ice fractions obtained from the first and second purification rounds were assessed using BCA concentration assay, SDS-PAGE, silver staining, fluorescence measurements, and mass spectrometry. Figures 6 and 7 present the fluorescence signals of eGFP-*Ri*AFP purified by FWIP. Following consecutive cycles of FWIP, the amount of IBP in the loading solution was reduced, leading to a reduction in the fluorescence of the ice cubes (Fig. 7). After one round of purification, the total protein (IBP + non IBP) was reduced to 15% of the protein content in the loading solution, indicating that most of the IBP was extracted by the ice. The yield of eGFP-*Ri*AFP from the first round was 83%. After the second round of FWIP, the total protein concentration was further reduced. The recovery after two rounds of purification was 76%. More impurities were present in the ice during the first round due to the higher concentration of the initial solution. The purity of *Tm*AFP after two rounds of FWIP was >95%, as verified by mass spectrometry (Supplementary Fig. S3) and TH measurements.

## Discussion

The FWIP method was developed for the efficient purification of large amounts of IBPs. As in other ice affinity purification methods, the FWIP method did not require protein affinity tags and is applicable to different types of IBP solutions, including hemolymph fluid. In all cases in which we used diluted solutions containing 0.16 mg/mL non-IBPs or less (Fig. 3), we obtained high-purity IBP solutions within a single round of FWIP. Reducing the dilution dramatically increases the amount of impurities in the ice fraction. This increase could be related to constitutional supercooling^28^ in which the growing front of ice develops fingering ice patterns that allow impurities to be incorporated into the ice. It might be that under the given rate of ice growth in this system the initiation of fingering growth is at a much lower concentration (mg/mL regime).

The yield that can be obtained from a single round could be increased by performing more FWIP cycles (Fig. 1), and the purity could be increased by applying additional rounds. The starting volume, starting concentration, and the number of cycles and rounds could be adjusted according to the total protein and impurity amounts present in the loading solution and the final purification level needed.

In the course of determining the effectiveness of the system, we found an interesting nonlinear correlation between the amount of impurities present in the loading solution and the amount of impurities incorporated into the ice. This phenomenon is related to the temperature profile near the ice surface and the flow rate of the water. These parameters could be physically manipulated if needed. A maximum concentration of 0.037 mg/mL type III AFP could be incorporated into the ice. This maximum level probably arose from the saturation of the IBPs on the ice and is a function of the IBP type, because different IBPs have different binding kinetics and different binding sites on ice^29^. This concentration corresponded to 4.5 μM, indicating a volume of 70 × 70 × 70 nm^3^ per molecule. Drori *et al*. performed accumulation experiments of IBP on ice surfaces which revealed that IBPs accumulated on stationary ice surfaces to reach surface densities of 7–35 nm between IBPs with a typical accumulation time of several seconds^30^. In our experiments, the ice cube grew to a thickness of 13 mm in 30 minutes; thus, the ice grew at a rate of approximately 7 μm/s. It is reasonable to assume that ice grew in layers on which the IBPs accumulated at the saturation level.

Our analysis of the IBP concentrations in the ice relative to the total protein concentration in the loading solution revealed that the amounts of non-IBPs present in the loading solution did not affect the amounts of IBPs extracted into the ice. This is an important fact when considering the reproducibility of the purification process. The insensitivity of the purification process to impurities relied on the fact that ice excludes solutes in a colligative manner.

FWIP is suitable for purification of both recombinant and native IBPs, however, like other ice affinity purification methods, it cannot separate different native isoforms of the specific type of IBP. FWIP is advantageous over other ice affinity methods because it is suitable for large-scale protein purification over short time frames. The largest KM machine available commercially by Hoshizaki produces 600 kg of ice in 24 h. With simple adjustments, this machine may be used to purify grams of IBPs per day. To concentrate the large amount of solution from the melted ice that includes pure IBPs (II fractions), tangential flow filtration (TFF) system can be used. For example, 300 L of solution can be concentrated in 24 hours by a commercially available membrane. The FWIP system does not require resins and does not produce chemical waste. These factors render the FWIP method readily suitable for IBP purification for use in the food and medical industries. The FWIP method can also be used for small-scale purification. We anticipate that the FWIP method has a potential to advance the practical use of IBPs in research and medicine.

## Materials and Methods

### Expression of recombinant type III AFP, MBP-*Tm*AFP, and eGFP-*Ri*AFP

A plasmid containing a type III AFP, QAE isoform from ocean pout, was obtained from Peter L. Davies (Queen’s University, Canada)^31^. A plasmid containing the recombinant eGFP-*Ri*AFP gene was generously donated by Aaron Hakim (Yale University, New Haven)^32^. A plasmid containing recombinant MBP-*Tm*AFP was obtained from the lab of Deborah Fass (The Weizmann Institute, Rehovot, Israel)^33^. A plasmid bearing MBP-*Tm*AFP was transformed using the heat shock method^34^ into the Origami-B *E. coli* strain (Novagen). All other plasmids were transformed by heat shock into BL21-DE3-PlysS *E. coli* strain. A stirred tank fermentor of 1 or 5 L (Applikon Biotechnology, The Netherlands) was used for a fed-batch fermentation process to produce all recombinant proteins. Bacteria were grown under controlled physical and chemical parameters (temperature, air flow, oxygen flow, agitation speed, foam formation, pH, and dissolved oxygen). The feed and medium components (e.g. carbon and nitrogen sources, salts) were supplied during the fermentation process by direct injection into the vessel. Air was supplied at a rate of 0.15–2 vvm (i.e. volume of air per volume of medium per minute) to maintain the dissolved oxygen concentration greater than 20% of air saturation. pH was maintained at 7 by the titration of either 2M H_2_SO_4_ or 3 M NaOH. The cultivation temperature was 37 °C and then lowered to 15–20 °C prior to induction with 1 mM of IPTG. Typical fermentation time duration was 20–24 hours, final OD values reached 60. Cells were harvested, pelleted by centrifugation at 4 °C at 4500 rpm for 45 min, and stored at −80 °C until purification. Five grams of wet cell pellet were resuspended in 50 mL 50 mM sodium phosphate buffer (pH 7.5) supplemented with protease inhibitors, precooled to 4 °C. The resuspended cells were lysed by sonication and centrifuged at 4 °C at 15000 rpm for 45 min. The supernatant of the lysate was diluted to 2.5 L and a final concentration of 25 mM of sodium phosphate buffer (pH 7.5, starting solution).

### Purification by FWIP

Two and a half liters of the crude starting solution were chilled to 4 °C and loaded in a pressure tank under a pressure of 2.5 bar. We slightly reduced the water flow to improve the efficiency of purification. The machine was then turned on and a cycle of ice formation was started, as explained in the results section. During the cycle, ~30% of the solution gradually froze. At the end of the cycle, before the defrost step, the ice cubes were subjected to dry air flow to remove residual liquid from the ice surface. We found that air was more effective than rinsing for this purpose. The ice cubes were collected and stored. During the second cycle, the unfrozen liquid (drain) was collected and diluted using double distilled water to reach the initial buffer concentration of 25 mM and a volume of 2.5 L. This solution was loaded into the pressure tank and the system was turned on again. After completing the desired number of cycles, all ice fractions were collected and mixed. The series of FWIP cycles was termed the first round of FWIP. To improve the protein purity, the ice collected from the first round was melted in a 20 °C water bath, and 1M sodium phosphate buffer was added to a final concentration of 25 mM. The melted ice was used as a starting solution for a second round of purification. A schematic diagram of the process is shown in Fig. 1.

### IBP purity assessment in the ice fractions

The type III AFP was purified by FWIP in previous experiments and a purity of >95% was verified using silver staining and thermal hysteresis measurements based on known activity values^31^. A total of 132 mg of type III AFP was diluted in a crude lysate solution that did not contain any IBP at a concentration of 0.16 mg/mL to a final volume of 2.5 L. We conducted 10 cycles of FWIP to extract all IBP from the solution, which gave 7 L of ice. The 10th cycle contained only 0.6 mg of protein, indicating that >99.5% of type III AFP had been extracted. We verified that the drain did not contain any AFPs using SDS-PAGE of the concentrated drain solution. The purity level was calculated by measuring the amount of total protein present in the ice fractions. A total of 147 mg of protein was present in the ice, 132 mg of which was type III AFP. The 15 mg of non-IBPs were impurities. Given that this amount was divided among 7 L, and that the amount of impurities remained constant in all cycles, as shown, 0.002 mg/mL of impurities were present in the final solution.

The SDS-PAGE and protein concentration analyses were conducted using samples obtained from the ice and drain fractions. The ice fraction sample was obtained by collecting 5 cubes of ice from 5 different rows of the metal plate to avoid possible bias due to temperature gradients on the metal plate. Equal volume samples (20–40 mL) of the fractions were collected from each cycle and were concentrated to the same volume using the Vivaspin 20 (Sartorius, UK). Large ice fractions up to 5 L were concentrated using a 2.5 L ultrafiltration stirred cell (Merck, Germany). Ice fractions of >5 L were concentrated using a TFF system (GE Healthcare, UK), with a 3 or 10 kDa cutoff. TFF systems can be obtained with a concentration rate capacity per hour that matches the amounts of ice produced by the ice machine.

### Bromophenol extraction

Two and a half liters of a 0.05% (w/v) bromophenol blue dye solution (750 µM) was loaded into the ice machine and allowed to run for one cycle. The ice obtained was analyzed by absorbance spectroscopy at 600 nm (GENESYS 10 VIS spectrophotometer, Thermo Fisher Scientific, USA).

### Cell lysate extraction that does not contain IBPs

The BL21-DE3-PlysS *E. coli* cells (not transformed with a plasmid bearing an IBP gene) were cultured in a fed-batch as described above. The soluble fraction obtained from a 16.5 g pellet was diluted to 2.5 L with a final concentration of 25 mM sodium phosphate buffer (pH 7.5). This starting solution contained 0.55 mg/mL proteins (note that the proteins do not include  IBPs). The starting solution was loaded in the ice machine and allowed to run. At the end of the process, all ice fractions were collected and melted. Samples from the starting solution, the ice, and the drain fractions were analyzed. The same process was repeated after dilution of the loading solution to 3.9 L, 5.3 L, 6.7 L, 8.1 L, and 11.5 L, giving diluted starting solutions with the same amount of proteins but at lower concentrations. The protein concentration of the fraction was measured using the micro BCA protein assay (described below).

### Isolation of *Tm*AFP

*Tenebrio molitor* larvae were treated as described previously^35^. Briefly, the larvae were acclimated for 1 month at 4 °C and stored at −80 °C. Ten grams of the larvae were crushed and homogenized in 100 mL of a 100 mM ammonium bicarbonate (pH 8) buffer supplemented with 0.1 M phenylthiocarbamide and protease inhibitors. The homogenate was centrifuged at 4 °C and 50,000 rpm (Sorvall WX 90 Thermo Electron Corporation, USA) for 1 h. The supernatant was diluted to a final volume of 2.5 L and final concentration of 25 mM ammonium bicarbonate (pH 8) and loaded into the pressure tank. After 4 cycles of purification, 60 mg of *Tm*AFP (including low amounts of impurities) were obtained in the ice fractions. After the second round of purification (4 cycles), a total of 45 mg *Tm*AFP were isolated. The protein content in the II fractions was assessed by mass spectrometry (Supplementary Fig. S3) and thermal hysteresis measurements due to the difficulties associated with identifying this protein by Coomassie blue staining^33^.

### Mass spectrometry

The protein samples were prepared on a ground steel MSP96 target (Bruker Daltonik, Germany) using HCCA (α-cyano-p-hydroxycinnamic acid Bruker Daltonik, Germany) as a matrix, according to the manufacturer’s recommendations. Protein Standard I (Bruker Daltonik GmbH, Germany; containing Insulin, Ubiquitin, Cytochrome C and Myoglobin) and Protein Standard II (Bruker Daltonik GmbH, Germany; containing Trypsinogen, Protein A and BSA) were used to calibrate the respective mass ranges. Mass spectrometry measurements were performed in a microflex MALDI-TOF (Bruker Daltonik GmbH, Germany). The spectra fell within the mass range 5–60 kDa.

### SDS-PAGE and protein concentration determination

SDS-PAGE was performed in a 6% stacking gel, 18% resolving gel following common procedures^36^. Protein concentration was determined using the Micro BCA Protein Assay (Thermo Scientific, USA) against a bovine serum albumin (BSA) standard. The absorbance was read at 550 nm using an ELx808 ultra microplate reader (BioTek Instruments, USA). Sodium phosphate buffer 25 mM was used as a blank.

### Fluorescence quantification of the eGFP-*Ri*AFP

The fluorescence was quantified using a Biotek Synergy-2 plate reader fluorometer (Cary Eclipse, with a polarized light accessory). The total protein concentration was determined using the Micro BCA Protein Assay (Thermo Scientific, USA) according to the manufacturer’s instructions, with the absorbance read at a wavelength of 562 nm. The eGFP-*Ri*AFP concentration was estimated using GFP fluorescence measurements with 485/20 nm excitation and 528/20 nm emission filters. All samples were concentrated to the same volume.

### Thermal hysteresis measurements

The thermal hysteresis measurements were conducted using a computer-controlled nanoliter osmometer as described elsewhere^37^.

### Data availability

Almost all data generated and analysed during this study are included in this published article (and its Supplementary Information files). All datasets are available from the corresponding author on reasonable request.

## Electronic supplementary material

Supplementary figures
