# Supplementary material for: Falling water ice affinity purification of ice-binding proteins
Source: Sci Rep. 2018 Jul 23;8:11046. doi: 10.1038/s41598-018-29312-x (PMC6056459; doi:10.1038/s41598-018-29312-x)
Supplement: Supplementary file 1 — Supplementary figures [file 41598_2018_29312_MOESM1_ESM.pdf]

Falling water ice affinity purification of ice-binding proteins

Chen Adar, Vera Sirotinskaya, Maya Bar Dolev, Tomer Friehmann, and Ido Braslavsky

Supplementary Materials:

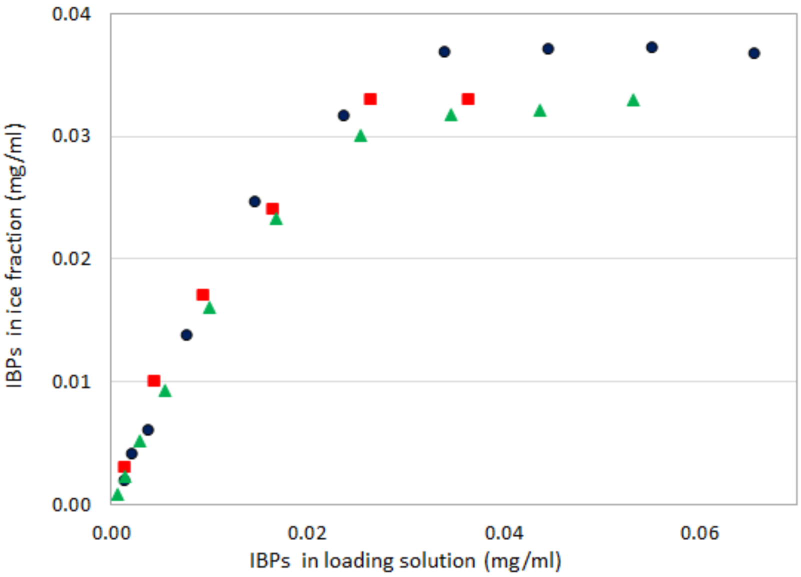

Supplementary Figure S1. The dependence of the IBP concentrations in the ice fractions on the IBP concentrations in the starting solution. The following symbols represent: (●) solutions of pure type III AFP, (■) lysate supernatant solution of *E. coli* expressing type III AFP, (▲) mixture of IBP-free *E. coli* lysate supernatant and pure type III AFP solutions.

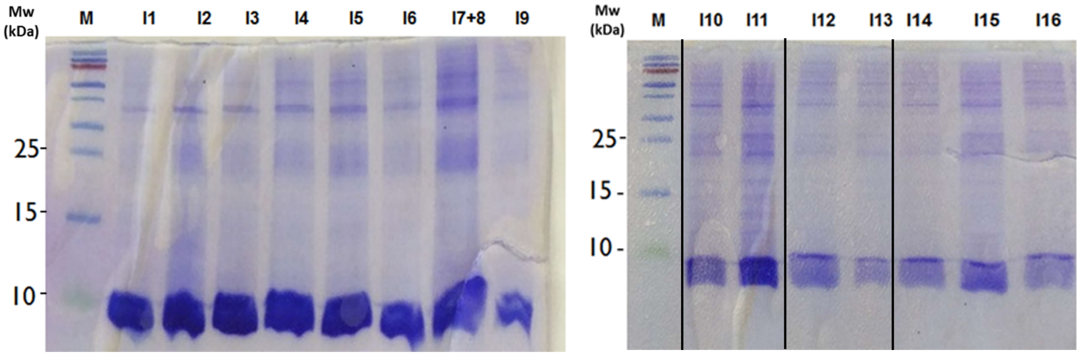

Supplementary Figure S2. SDS-PAGE analysis of the type III AFP purified by FWIP. A 19 g pellet was treated as described to obtain 9.5 L of the loading solution and was allowed to run in the ice machine for 16 cycles (first purification round). The samples from all ice fractions were concentrated to equal volumes; the gels were processed in parallel. Although the quantity of AFP decreased during this process, the quantities of impurities present remained constant. The gels were cropped, the full-length gels are presented in Supplementary Figure S4.

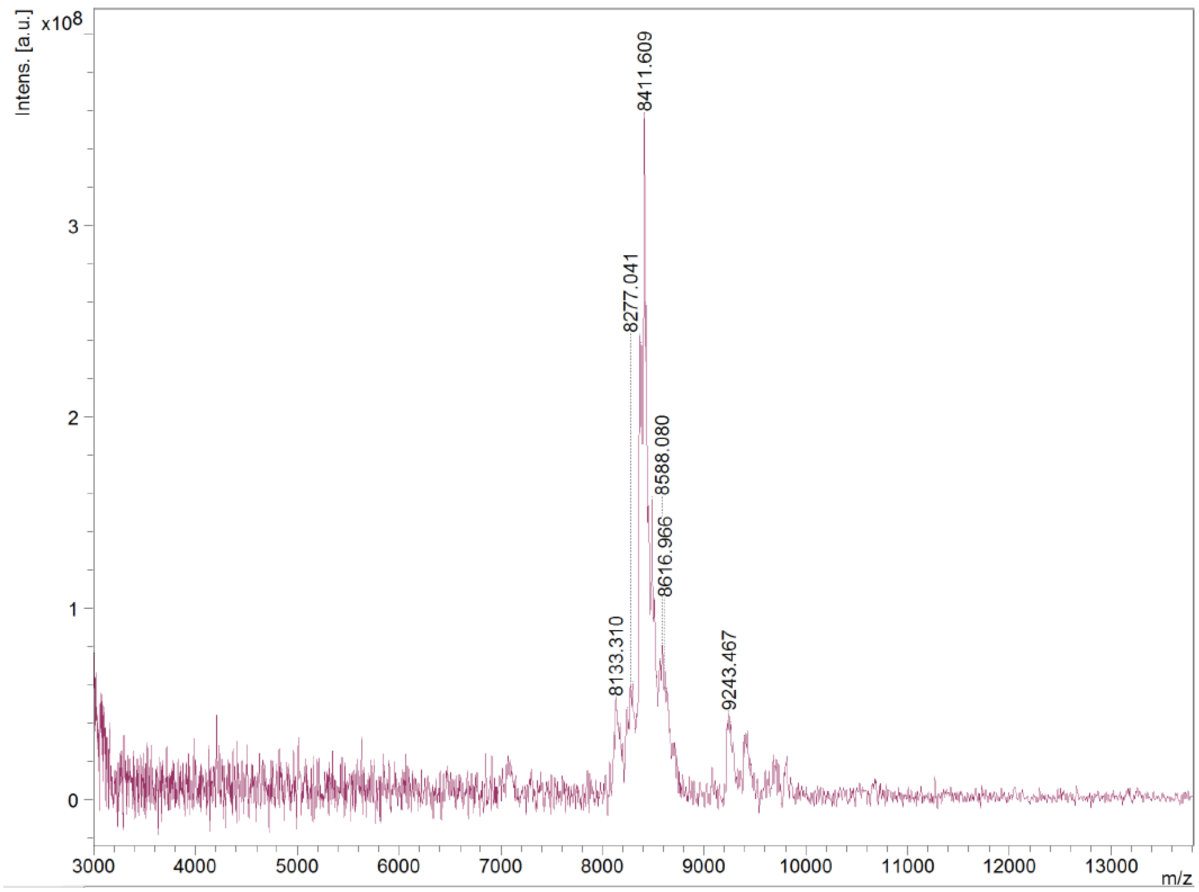

Supplementary Figure S3. Mass spectrometry analysis of *Tm*AFP after two rounds of FWIP (II fractions). The peaks represent *Tm*AFP and its isoforms. No significant peaks were observed above 13,000 Da.

a

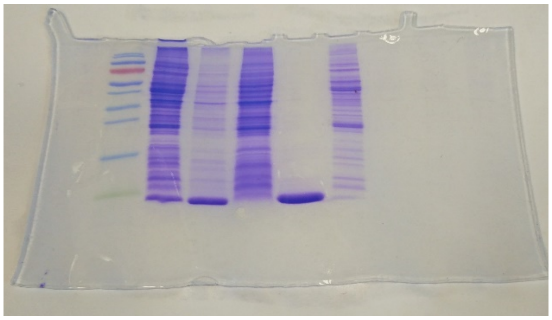

b

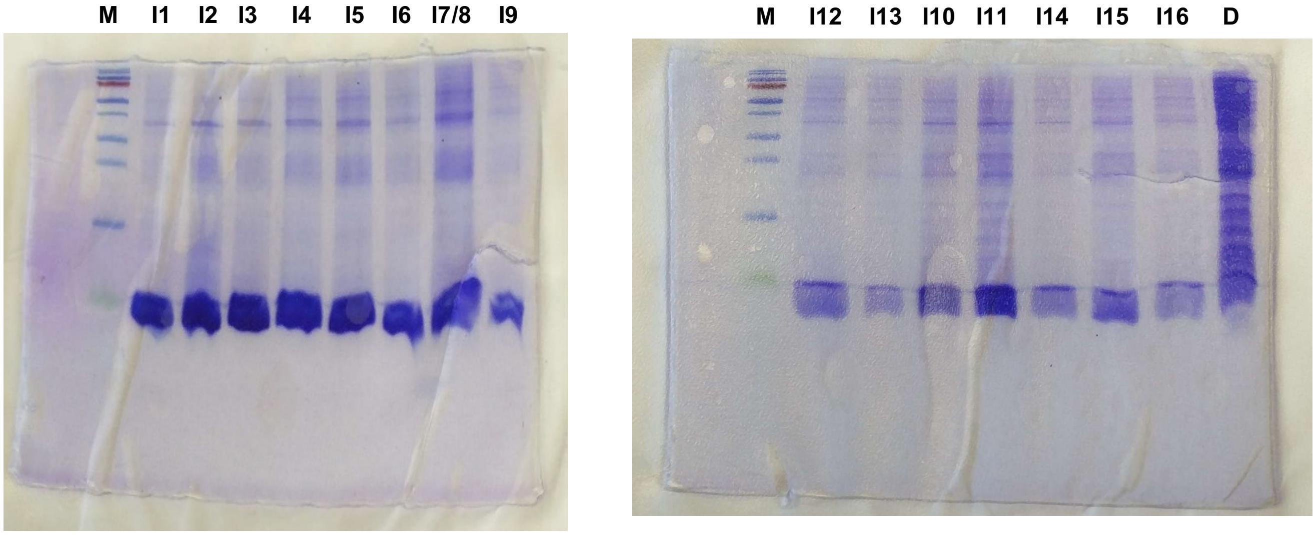

Supplementary Figure S4. Uncropped images of electrophoretic gels (**a**) Figure 5 (**b**) Supplementary Figure S2.
